# A *Streptomyces coelicolor* host for the heterologous expression of Type III polyketide synthase genes

**DOI:** 10.1186/s12934-015-0335-0

**Published:** 2015-09-16

**Authors:** Anyarat Thanapipatsiri, Jan Claesen, Juan-Pablo Gomez-Escribano, Mervyn Bibb, Arinthip Thamchaipenet

**Affiliations:** Department of Genetics, Faculty of Science, Kasetsart University, Bangkok, 10900 Thailand; Department of Molecular Microbiology, John Innes Centre, Norwich, NR4 7UH UK; Center for Advanced Studies in Tropical Natural Resources, NRU–KU, Kasetsart University, Bangkok, 10900 Thailand; Department of Bioengineering and Therapeutic Sciences and the California Institute for Quantitative Biosciences, University of California San Francisco, San Francisco, CA 94158 USA

**Keywords:** *Streptomyces*, Expression host, Type III polyketide synthases

## Abstract

**Background:**

Recent advances in genome sequencing, combined with bioinformatic analysis, has led to the identification of numerous novel natural product gene clusters, particularly in actinomycetes of terrestrial and marine origin. Many of these gene clusters encode uncharacterised Type III polyketide synthases. To facilitate the study of these genes and their potentially novel products, we set out to construct an actinomycete expression host specifically designed for the heterologous expression of Type III PKS genes and their gene clusters.

**Results:**

A derivative of *Streptomyces coelicolor* A3(2) designed for the expression of Type III polyketide synthase (PKS) genes was constructed from the previously engineered expression strain *S. coelicolor* M1152 [Δ*act* Δ*red* Δ*cpk* Δ*cda rpoB*(C1298T)] by removal of all three of the endogenous Type III PKS genes (*gcs,srsA,rppA*) by PCR targeting. The resulting septuple deletion mutant, M1317, proved to be an effective surrogate host for the expression of actinobacterial Type III PKS genes: expression of the reintroduced *gcs* gene from *S. coelicolor* and of the heterologous *rppA* gene from *Streptomyces venezuelae* under the control of the constitutive ermE* promoter resulted in copious production of germicidin and flaviolin, respectively.

**Conclusions:**

The newly constructed expression host *S. coelicolor* M1317 should be particularly useful for the discovery and analysis of new Type III polyketide metabolites.

## Background

Type III polyketides are generally small molecules generated by Type III polyketide synthases (PKSs). These multifunctional ketosynthases typically utilise free CoA thioesters as substrates without the involvement of an acyl carrier protein (ACP), and frequently catalyse specific cyclization reactions found in aromatic and pyrone polyketides [1, 2]. The recent explosion in genome sequencing, combined with bioinformatic analysis, has led to the identification of numerous uncharacterized Type III PKS genes in plants, fungi and bacteria, including actinomycetes [1, 3–6]. Bacterial Type III PKSs are attractive to study for a number of reasons: they are widely distributed, make a variety of structurally different products and, compared to Type I and Type II PKSs, relatively simple to manipulate genetically [7]. Some Type III PKSs are solely responsible for product formation. For example, germicidin synthase (Gcs) and the 1,3,6,8-tetrahydroxynaphthalene (THN) (RppA) synthases, commonly found in *Streptomyces* spp., are the only enzymes required to produce germicidin and THN, respectively [5, 8, 9]. THN can oxidise spontaneously to yield the red-brown pigment flaviolin, which is involved in bacterial melanin production, although a cytochrome P450 monooxygenase was reported to be involved in the conversion of THN to flaviolin in *Streptomyces antibioticus* [6, 10, 11]. Some Type III PKS genes are organized in small operons, such as *srsABC* of *Streptomyces griseus* that produces alkylresorcinols [12]. Such operons may also reside in complex gene clusters where they provide a specific precursor for the biosynthesis of more elaborate specialized metabolites; for example, the *dpgABCD* operon that synthesizes dihydroxyphenylacetic acid which is used as a precursor for vancomycin, balhimycin, teicoplanin and kendomycin biosynthesis in *Amycolatopsis orientalis* [13], *A. balhimycina* [14], *Actinoplanes teichomyceticus* [15] and *Streptomyces violaceoruber* [16], respectively.

Within the actinomycetes, *Streptomyces* species such as *Streptomyces lividans* TK24, *Streptomyces coelicolor, Streptomyces avermitilis* and *Streptomyces albus* J1074 [17, 18] have been used as surrogate hosts for the heterologous expression of gene clusters encoding specialized metabolites. *S. avermitilis*, the industrial producer of avermectin [19, 20], and *S. coelicolor*, for many years the model species for the study of the genetics and biology of actinomycetes [21], have both been manipulated genetically to enhance the level of expression of heterologous gene clusters [22–25], and derivatives of each have been used to produce a range of different heterologous specialized metabolites [23, 24, 26–32]. In this work, we have further engineered *S. coelicolor* M1152 [24], a derivative of strain M145 from which four of the known antibiotic biosynthetic gene clusters had been deleted and which contains a mutation (C1298T) in *rpoB* that increases the level of secondary metabolite production, by removing all of its native Type III PKS genes. Deletion of the resident Type III PKS genes could potentially increase precursor pool levels and prevent undesirable interference with biosynthesis encoded by heterologous Type III PKS genes.

## Results and discussion

### Type III PKS genes in *S. coelicolor*

**Fig. 1:**
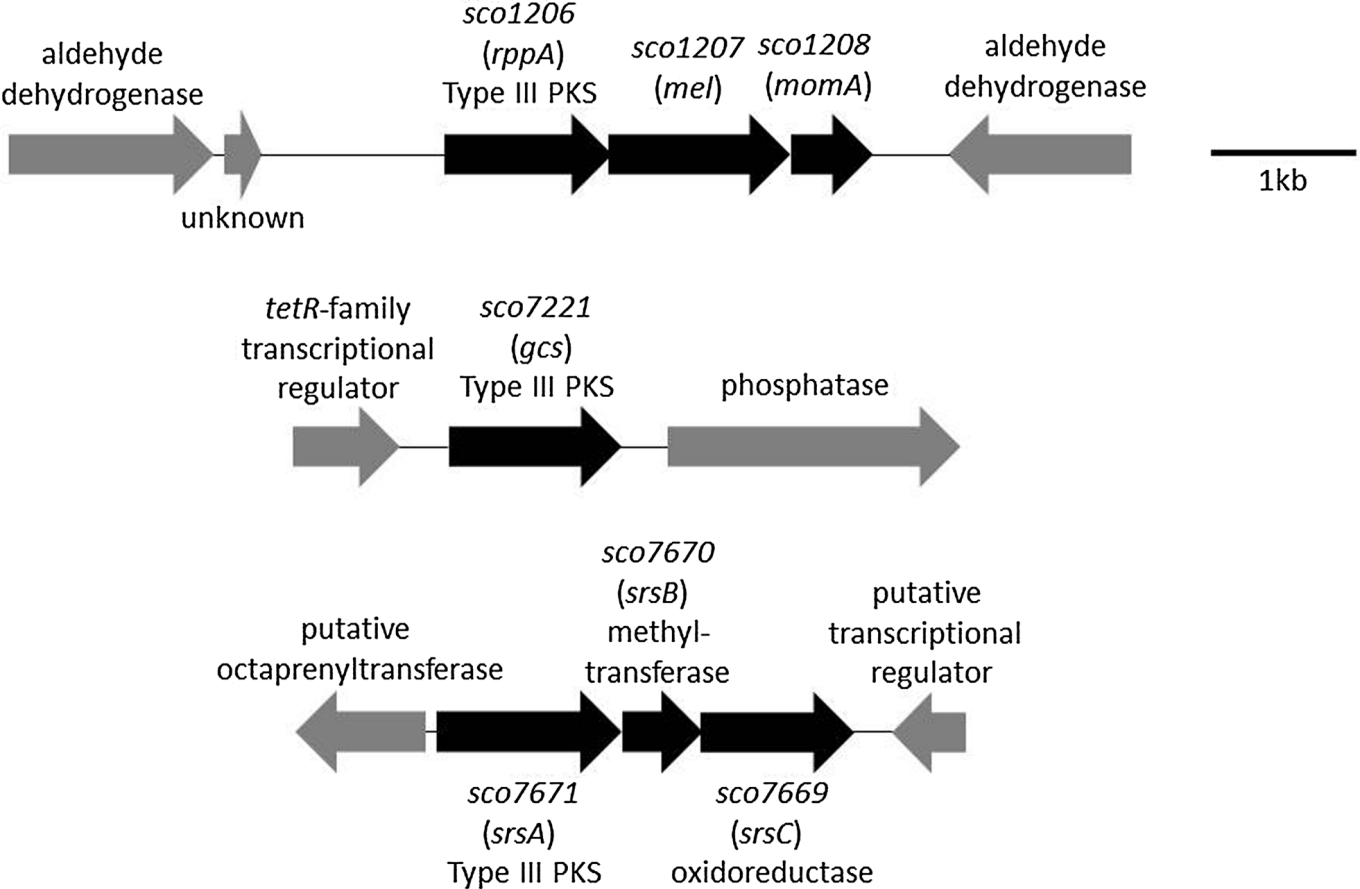
Genetic organisation of the three Type III PKS loci in *S. coelicolor. sco7221* (*gcs*), *sco1206*-*1207*-*1208* (*rppA* operon) and *sco7669*-*7670*-*7671* (*srs* operon) targeted for deletion are shown as *black arrows*. Flanking genes are shown in *grey* together with their putative functions

Analysis of the genome sequence of *S. coelicolor* M145 revealed three endogenous Type III PKS genes: *sco1206*, *sco7221* and *sco7671* [33]. *sco1206* encodes RppA which synthesizes 1,3,6,8-tetrahydroxynaphthalene (THN), the intermediate involved in bacterial melanin biosynthesis, by the condensation of five malonyl CoA molecules [8]. *sco1206* lies upstream of *sco1207* and *sco1208* (Fig. 1), which are homologues of the characterized cytochrome P450 genes *mel* [6] and *momA* [10] in *S. griseus* and *S. antibioticus*, respectively; in each of these species, the single P450 gene lies adjacent to a *rppA* homologue. *S. coelicolor* thus differs in possessing two tandemly arranged P450 genes 3′ of the Type III PKS gene, with all three genes apparently translationally coupled. MomA was predicted to play a role in the oxidation of THN to form flaviolin [10], while Mel was thought to couple molecules of THN to generate hexahydroxyperylenequinone melanin [6]. Since *S. coelicolor* is not known to produce any of these three molecules, this gene cluster may not be expressed under commonly used laboratory growth conditions. *sco7221* was shown to play a role in germicidin biosynthesis [5, 34] and its product thus designated as germicidin synthase (Gcs). Analysis of Sco7671 revealed closest homology to a family of mycobacterial Type III PKSs [34]. *sco7671, sco7669* and *sco7670* are likely to form an operon (Fig. 1) organized in a similar fashion to *srsABC* of *S. griseus* (known to produce phenolic lipids, the alkylresorcinols) [12]. Although the function, substrate specificity and the product profile of *sco7671* have been investigated in vitro [34], the final product of the gene cluster has not been identified. To generate a clean host for the heterologous expression of Type III PKS gene clusters, we set out to delete *sco7221* (*gcs*), *sco7669*-*7670*-*7671* (the *srs* operon) and *sco1206*-*1207*-*1208* (the *rppA* operon) from *S. coelicolor* M1152 [24].

### Deletion of the three native Type III PKS genes and operons of *S. coelicolor* M1152

**Fig. 2:**
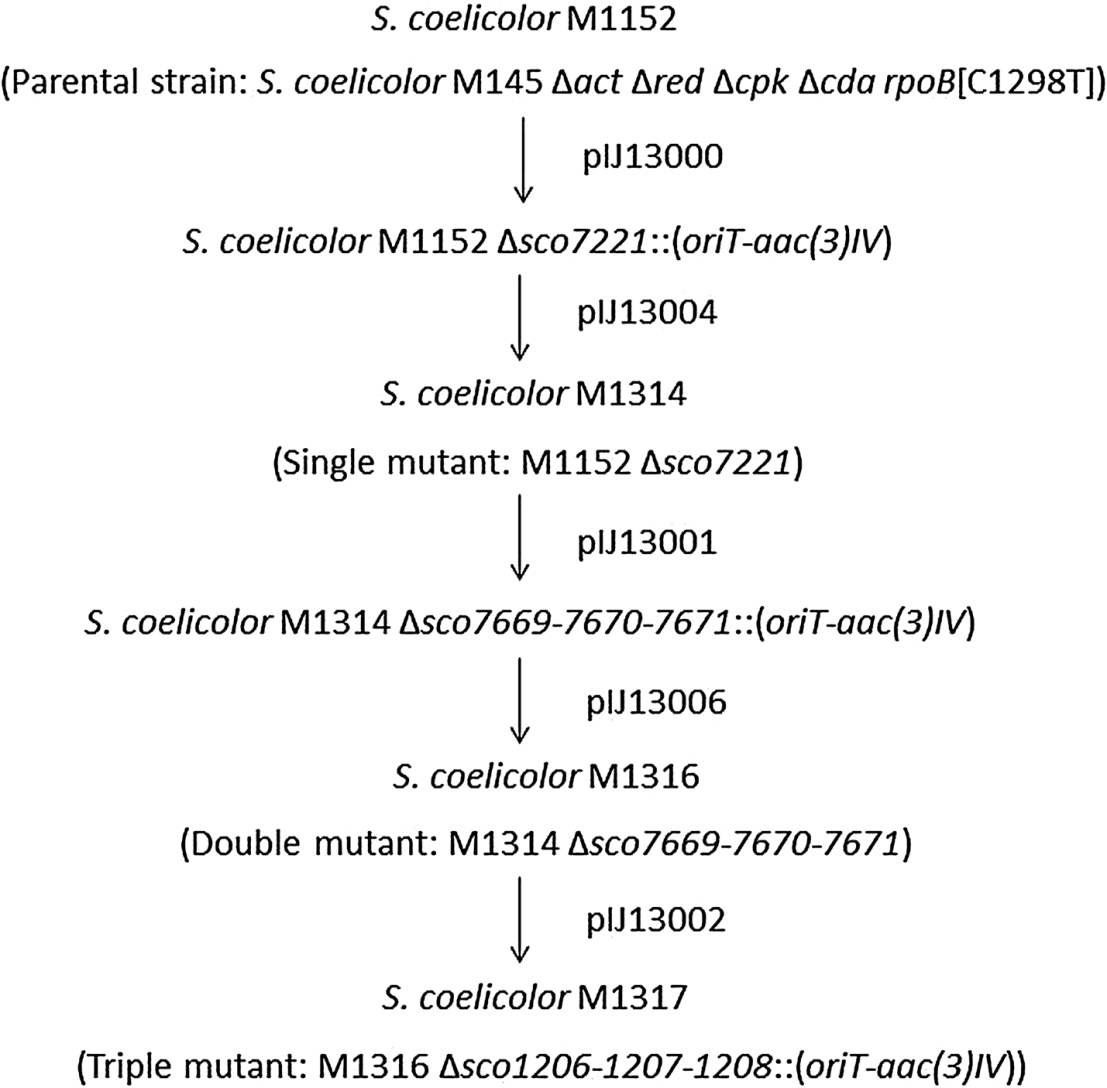
Construction of Type III PKS deletion mutants of *S. coelicolor* M1152. Mutagenized cosmids introduced into each strain are indicated by their pIJ numbers

All three Type III PKS genes and operons were deleted from *S. coelicolor* M1152 by PCR targeting [35, 36] (Fig. 2; see reference 35 for a detailed description of PCR targeting). For two of the mutations (Δ*sco7221* and Δ*sco7669*-*7670*-*7671*), in-frame deletions were generated in a two-step process; in the first step, the entire gene or operon in the corresponding cosmid was replaced with an apramycin (Apr) resistance cassette in *Escherichia coli* and the mutant allele then introduced into *S. coelicolor* M1152 to create an Apr^R^-marked mutant; in the second step, the Apr^R^ cassette in the cosmid was eliminated in *E. coli* using FLP-recombinase to leave an 81 bp scar sequence which was then introduced into the Apr^R^-marked mutant, replacing the Apr^R^ cassette and creating an in-frame deletion mutant. Deletion of *gcs* (*sco7221*; 1122 bp) yielded M1314, which was then used for the in-frame deletion of the *srs* operon (*sco7669*-*7670*-*7671*; 2786 bp) to yield the double Type III PKS deletion mutant, M1316. Finally, the *rppA* operon (*sco1206*-*1207*-*1208*; 2859 bp) was removed from M1316 by replacement with the Apr^R^ cassette to yield the triple mutant M1317 (Fig. 2). Confirmation of the mutations in M1314, M1316 and M1317 was achieved by PCR amplification using primers flanking the deleted regions of the chromosome followed by DNA sequencing (data not shown). With the previous removal of four antibiotic biosynthetic gene clusters (to generate M1152), the resulting septuple mutant M1317 has lost approximately 178 kb of chromosomal DNA compared to the parental strain M145.

All three deletion mutants grew and sporulated in a manner that was indistinguishable from M1152 when cultures were grown on SFM agar (data not shown).

### Validation of *S. coelicolor* M1317 as a host for the expression of Type III PKS genes

**Fig. 3:**
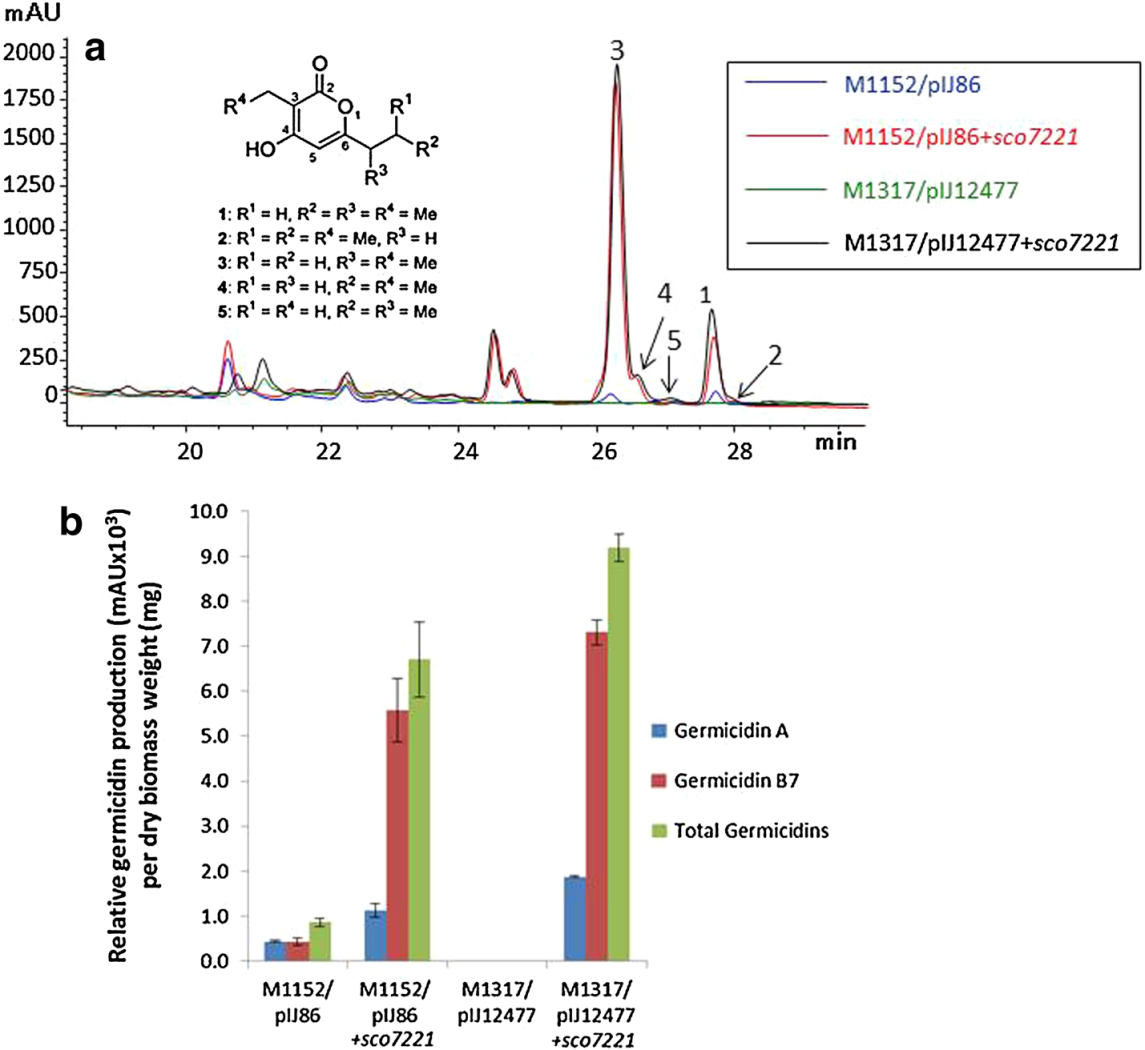
Germicidin production in derivatives of *S. coelicolor* M1152. **a** HPLC chromatogram and chemical structures of germicidin derivatives [5]. Mass spectroscopy confirmed the production of the following five germicidin congeners: 1, [M + H]^+^ = 197 *m*/*z*; 2, [M + H]^+^ = 197 *m*/*z*; 3, [M + H]^+^ = 183 *m*/*z*; 4 [M + H]^+^ = 183 *m*/*z* and 5, [M + H]^+^ = 183 *m*/*z*. The HPLC profiles for M1152 and M1317 were indistinguishable from those for M1152/pIJ86 and M1317/pIJ12477, respectively (data not shown). **b** Germicidin A, germicidin B7, and total germicidin production per mg dry weight of mycelium. The histograms show the average values obtained from duplicate cultures with the two individual measurements (Table 1) indicated by *bars*

The well-characterized germicidin synthase gene of *S. coelicolor* was chosen initially to validate the utility of M1317 as a host for the expression of Type III PKS genes. *gcs* (*sco7221*), which is capable of producing five congeners [5], was inserted downstream of the constitutive ermE* promoter in two bifunctional multi-copy vectors, pIJ86 and pIJ12477 (a derivative of pIJ86 conferring kanamycin resistance), to give pIJ86 + *sco7221* and pIJ12477 + *sco7221*, respectively. pIJ86 + *sco7221* and pIJ86 were then transferred by conjugation into M1152 to yield M1152/pIJ86 + *sco7221* and M1152/pIJ86, respectively, while pIJ12477 + *sco7221* and pIJ12477 were transferred similarly to M1317 to obtain M1317/pIJ12477 + *sco7221* and M1317/pIJ12477, respectively. Germicidin production was assessed by HPLC analysis of 5 days culture supernatants of all four strains (Fig. 3a), and the masses of the peaks corresponding to each germicidin congener [5] confirmed by mass spectrometry. While no production was observed, as expected, in M1317/pIJ12477, peaks corresponding to most of the germicidin congeners were detected in culture supernatants from the other three strains, with total production levels in M1317/pIJ12477 + *sco7221* and M1152/pIJ86 + *sco7221* 10.7- and 7.8-fold higher than in M1152/pIJ86, presumably at least partly attributable to the multi-copy nature of the vectors; production in M1317/pIJ12477 + *sco7221* was 1.4-fold higher than in M1152/pIJ86 + *sco7221*, potentially reflecting increased precursor supply in the septuple deletion mutant. In each case, germicidin A and germicidin B7 were the major products (Fig. 3; Table 1).

**Table 1:** Relative levels of production of germicidins and flaviolin in engineered strains

Strain	Relative production (mAU × 10^3^)/mg dry weight			
	Germicidin A	Germicidin B7	Total germicidins	Flaviolin
M1152/pIJ86	0.44 ± 0.02	0.42 ± 0.08	0.86 ± 0.10	None
M1317/pIJ12477	None	None	None	None
M1152/pIJ86 + *sco7221*	1.13 ± 0.14	5.58 ± 0.70	6.71 ± 0.84	ND
M1317/pIJ12477 + *sco7221*	1.88 ± 0.03	7.32 ± 0.27	9.19 ± 0.30	ND
M1152/pIJ12477 + *sven5367*	ND	ND	ND	1.72 ± 0.27
M1317/pIJ12477 + *sven5367*	ND	ND	ND	2.16 ± 0.05

*ND* not determined

**Fig. 4:**
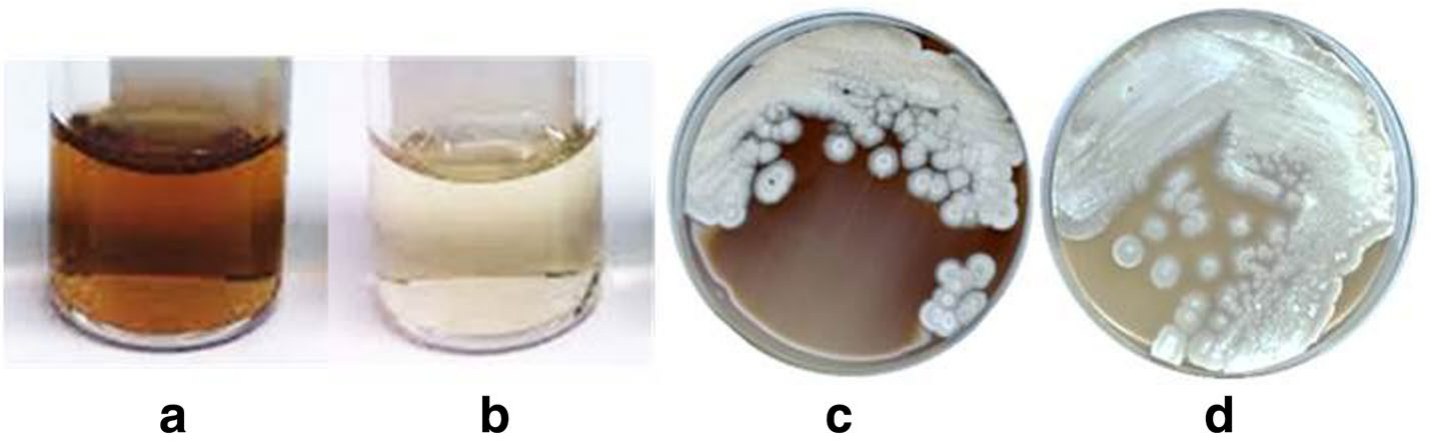
*Red-brown* pigment production after heterologous expression of the *rppA* homologue of *S. venezuelae* in *S. coelicolor* M1317/pIJ12477 + *sven5367* (**a**, **c**) compared to the same host containing the empty expression vector (M1317/pIJ12477) (**b**, **d**). **a**, **b** cultures grown in GYM liquid medium; **c**, **d** cultures grown on IPM agar

**Fig. 5:**
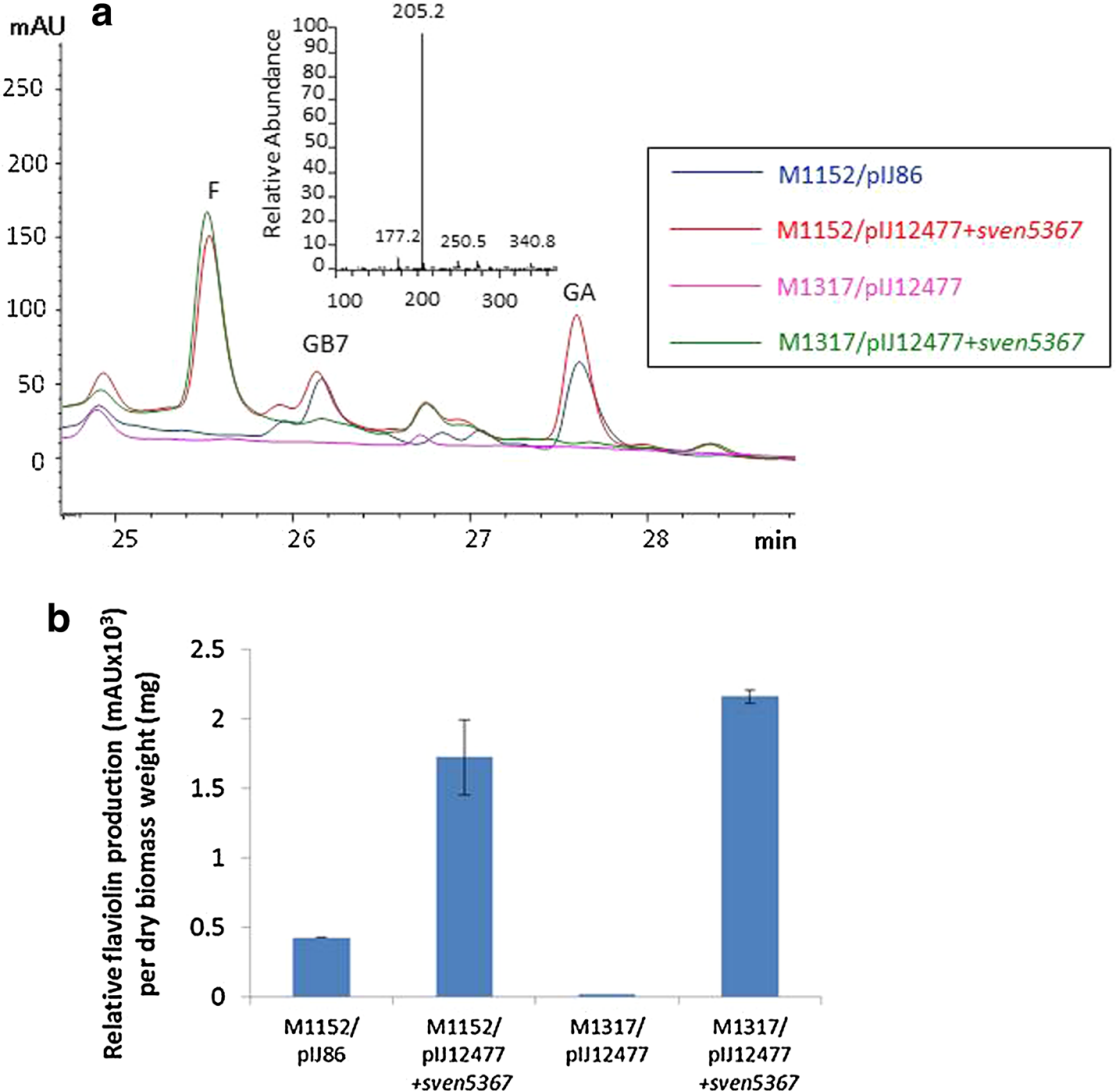
Flaviolin production in derivatives of *S. coelicolor* M1152. **a** HPLC chromatogram of flaviolin (F) production in M1152/pIJ12477 + *sven5367* and M1317/pIJ12477 + *sven5367*; in each case, a mass of [M + H]^−^ 205 *m/z* was confirmed by mass spectrometry (data for M1317/pIJ12477 + *sven5367* is shown in the *inset*). GA and GB7 indicate peaks of germicidin A and germicidin B7, respectively. The HPLC profiles for M1152 and M1317 were indistinguishable from those for M1152/pIJ86 and M1317/pIJ12477, respectively (data not shown). **b** Flaviolin production per mg dry biomass weight. The histograms show the average values obtained from duplicate cultures with the two individual measurements (Table 1) indicated by the *bars*

To evaluate the use of M1317 as a host for the heterologous expression of Type III PKS genes, the *rppA* homologue of *S.venezuelae* (*sven5367*) was cloned in pIJ12477 to yield pIJ12477 + *sven5367* which was introduced into M1152 and M1317 to produce M1152/pIJ12477 + *sven5367* and M1317/pIJ12477 + *sven5367*, respectively. Both strains produced a red-brownish pigment on IPM agar and in GYM liquid medium that was not observed in strains carrying the vectors alone (M1152/pIJ86 and M1317/pIJ12477) (Fig. 4). RppA initially generates 1,3,6,8-tetrahydroxynapthalene (THN) which is rapidly converted to flaviolin [6, 8]. Since we were unable to detect THN by chromatography, we instead quantified flaviolin production. The mass of the HPLC peak corresponding to flaviolin was confirmed by mass spectrometry [37] (Fig. 5a). Flaviolin production was readily detected in both M1152/pIJ12477 + *sven5367* and M1317/pIJ12477 + *sven5367*, while none was detected in M1152/pIJ86 and M1317/pIJ12477 (Fig. 5b; Table 1), thus establishing the utility of M1317 as a host for the heterologous expression of Type III PKS genes. Production in M1317/pIJ12477 + *sven5367* was 1.3-fold higher than in M1152/pIJ12477 + *sven5367*, again potentially reflecting increased precursor supply in the septuple deletion mutant. While some bacterial Type III PKSs, like plant chalcone and stilbene synthases [7], are sufficient alone for final product formation (e.g. the germicidin synthase of *S. coelicolor* [5] and the THN synthases of several *Streptomyces* species [8, 38, 39]), some require a cytochrome P450 encoded by a neighbouring gene to convert the intermediate they produce into the final product, e.g. the THN synthase of *S. antibioticus* [10]. Interestingly, while *rppA* of *S. venezuelae* lies adjacent to a gene encoding a cytochrome P450 that may be required for flaviolin biosynthesis in that strain, expression of *rppA* alone was all that was required for heterologous production of flaviolin in *S. coelicolor*. It is possible that in this strain THN is either oxidized spontaneously to flaviolin or oxidized by one of the remaining 18 P450 monooxygenases encoded by the *S. coelicolor* genome [40].

Our inability to detect flaviolin production in M1152/pIJ86 is consistent with previous work that also suggested that *sco1206* is not expressed in *S. coelicolor* under standard laboratory conditions [8]. However, when the *rppA* operon of *S. coelicolor* (*sco1206*-*1207*-*1208*) expressed from the ermE* promoter was introduced into M1317 to yield M1317/pIJ12477 + *sco1206*-*1207*-*1208*, red-brown pigment production occurred in both liquid- and agar-grown cultures. Flaviolin production in M1317/pIJ12477 + *sco1206*-*1207*-*1208* was confirmed by HPLC analysis and mass spectrometry (data not shown).

## Conclusions

We have successfully engineered the expression host *S. coelicolor* M1152 by removing all three of the endogenous Type III PKS gene and operons, and validated the resulting septuple deletion M1317 as a host for the ectopic and heterologous expression of Type III PKS genes. We believe that *S. coelicolor* M1317 will prove to be a useful strain for the future analysis of Type III PKS genes and their products from a wide range of actinomycetes.

## Methods

### Bacterial strains, plasmids, cosmids and culture conditions

Bacterial strains, plasmids and cosmids used and generated in this study are listed in Table 2. *Escherichia coli* strains were grown and manipulated following standard methods [35, 36, 41]. Media for the growth of *S. coelicolor* strains were: instant potato mash (IPM) agar [42], SFM agar [43], tryptic soy broth (TSB) and glucose-yeast extract-malt extract (GYM) medium [44]. Liquid cultures of *Streptomyces* strains were performed in 50 ml of medium in 250 ml flasks containing stainless steel springs at 30 °C with shaking at 250 rpm. The following antibiotics were used when required: 50 µg/ml apramycin (Apr), 50 µg/ml kanamycin (Kan), 50 µg/ml hygromycin (Hyg) and 25 µg/ml nalidixic acid (Nal). An overnight culture of each *Streptomyces* strain was used to inoculate 50 ml of medium to give an initial OD_600_ nm = 0.1.

**Table 2:** Bacterial strains, plasmids and cosmids used and generated in this study

Strain/plasmid/cosmid	Description	Source of reference
Strain		
*E. coli* BT340	DH5α/pCP20	[45]
*E. coli* BW25113	K-12 derivative: Δ*araBAD*, Δ*rhaBAD*	[46]
*E. coli* DH5α	For cloning and propagation of host strain, *deoR recA1 endA1 hsdR17*(*rk*-*, mk* ^+^) *phoA supE44 thi*-*1 gyrA96 relA1 λ* ^−^	Invitrogen, USA
*E. coli* ET12567	*dam*-*13::Tn9* *dcm*-*6* *hsdM* *hsdS*	[47]
*S. coelicolor* M1152	Derivative of *S. coelicolor* M145, Δ*act* Δ*red* Δ*cpk* Δ*cda* *rpoB*(C1298T)	[28]
*S. coelicolor* M1314	*S. coelicolor* M1152 Δ*sco7221*	This study
*S. coelicolor* M1316	*S. coelicolor* M1152 Δ*sco7221* Δ*sco7669*-*7670*-*7671*	This study
*S. coelicolor* M1317	*S. coelicolor* M1152 Δ*sco7221* Δ*sco7669*-*7670*-*7671* Δ*sco1206*-*1207*-*1208*::(*oriT*-*aac(3)IV*)	This study
Plasmid		
pGEM^®^-T Easy	*E. coli* vector for cloning PCR product, amp^r^	Promega, USA
pIJ86	*ermE*p*, *aac(3)IV*, *oriT* (RK2), *ori* (pIJ101)*, ori* (pUC18)	[48]
pIJ773	pBluescript KS (+), *aac*(*3*)*IV*, *oriT* (RK2), FRT sites	[35]
pIJ790	λ-RED (*gam*, *bet*, *exo*), *cat*, *araC*, *rep101* ^ts^	[35]
pIJ10701	pBluescript KS (+), *hyg*, *oriT* (RK2), *bla*	[35]
pIJ12477	pIJ86, *neo*	[49]
pIJ13000	St2H12 ∆*sco7221*::(*oriT*-*aac*(*3*)*IV*)	This study
pIJ13001	St4C2 ∆*sco7669*-*7670*-*7671*::(*oriT*-*aac*(*3*)*IV*)	This study
pIJ13002	2StG58 ∆*sco1206*-*1207*-*120*8::(*oriT*-*aac*(*3*)*IV*)	This study
pIJ13003	St2H12 ∆*sco7221*	This study
pIJ13004	pIJ13003::(*oriT*-*hyg*)	This study
pIJ13005	St4C2 ∆*sco7669*-*7606*-*7671*	This study
pIJ13006	pIJ13005::(*oriT*-*hyg*)	This study
pIJ13007	2StG58 ∆*sco1206*-*1207*-*1208*	This study
pIJ13008	pIJ13007::(*oriT*-*hyg*)	This study
pUZ8002	Kan^r^ */tra, neo, RP4*	[50]
Cosmid		
2StG58	SuperCos1 containing *sco1206*-*1207*-*1208*	John Innes Centre, UK
St2H12	SuperCos1 containing *sco7221*	John Innes Centre, UK
St4C2	SuperCos1 containing *sco7669*-*7670*-*7671*	John Innes Centre, UK
Sv3G11	SuperCos1 containing *sven5367*	John Innes Centre, UK
SuperCos1	*neo*, *bla*	Agilent Technology, USA

### DNA manipulations for *E. coli* and *Streptomyces*

Extraction of DNA from *E. coli* was carried out using standard methods [36] or by using a QIAprep Spin Miniprep Kit (QIAGEN) and following the manufacturer’s instructions. Genomic DNA was isolated from *Streptomyces* strains using the modified Kirby Mix procedure [43]. PCR amplifications for confirmation of engineered cosmids, mutant strains and expression constructs were performed using *Taq* DNA polymerase (QIAGEN). Expand High Fidelity PCR System (Roche) was used to amplify disruption cassettes [35] and Phusion^®^ High-Fidelity DNA Polymerase (NEB) was used to amplify PCR products for gene expression.

### Deletion of the Type III PKS genes of *S. coelicolor*

The three native Type III PKS genes and operons of *S. coelicolor* (*sco7221*, *sco7669*-*7670*-*7671* and *sco1206*-*1207*-*1208*) were deleted from strain M1152 [24] by PCR targeting [35, 41]. Three pairs of flanking primers, SCO7221FSpe and SCO7221RNhe, SCO7671FSpe and SCO7669RNhe, and SCO1206FSpe and SCO1208RNhe (Table 3), were designed to amplify a 1458 bp fragment carrying an apramycin resistance (Apr^R^) *oriT* cassette from pIJ773 [35]. Each of the Apr^R^ cassettes were flanked by 39 nt sequences matching the ends of the gene or operon to be deleted, and the targeted regions replaced with the Apr^R^ cassette by homologous recombination between these 39 nt sequences. Cosmids St2H12, St4C2 and 2StG58, containing *sco7221*, *sco7669*-*7670*-*7671* and *sco1206*-*1207*-*1208*, respectively, were transferred into *E. coli* BW25113 [47]/pIJ790 [35] and the resulting strains were then transformed with the corresponding replacement cassette. The mutagenized cosmids pIJ13000, pIJ13001 and pIJ13002, respectively, were verified by restriction enzyme digestion and PCR amplification with primers SCO7221TF and SCO7221TR, SCO7671TF and SCO7669TR, and SCO1206TF and SCO1208TR, respectively (Table 3). pIJ13000 (∆*sco7221*::(*oriT*-*aac*(*3*)*IV*)) was then introduced into *S. coelicolor* M1152 by conjugation with selection for apramycin resistance; subsequent screening for loss of kanamycin resistance (encoded by the SuperCos 1 vector) but retention of apramycin resistance was used to isolate the required ∆*sco7221*::(*oriT*-*aac*(*3*)*IV*) replacement mutant. In the meantime, the Apr^R^ cassettes were removed from pIJ13000, pIJ13001 and pIJ13002 in *E. coli* BT340 [46] using the FLP-recombinase, in each case leaving a 81 bp scar sequence in place of the cassette. The resulting non-transmissible cosmids, pIJ13003, pIJ13005 and pIJ13007, respectively, were confirmed by restriction enzyme digestion and PCR amplification using the same primer sets that were used for verification of the Apr^R^ cassettes (Table 3). To allow transfer of these cosmids from *E. coli* to *Streptomyces* via conjugation, a *bla*-*oriT*-*hyg*-*bla* cassette was amplified from pIJ10701 [35] and used to target the *bla* gene in the SuperCos1 backbone of pIJ13003, pIJ3005 and pIJ13007, yielding pIJ13004, pIJ13006 and pIJ13008, respectively. Intergeneric conjugation between the *S. coelicolor* ∆*sco7221*::(*oriT*-*aac*(*3*)*IV*) replacement mutant and *E. coli* ET12567/pUZ8002 [47, 50] containing pIJ13004 was performed as previously described [35, 41]. Exconjugants were selected based on hygromycin resistance and the desired ∆*sco7221* in-frame deletion mutant M1314 obtained by screening for Apr, Kan and Hyg sensitive segregants and confirmed by sequencing of the PCR product obtained from genomic DNA using primers SCO7221TF and SCO7221TR. Essentially the same procedure was used to generate the double in-frame deletion mutant M1316 (Δ*sco7221*, Δ*sco7669*-*7671*) from M1314 using pIJ13001 and pIJ13006 (and confirmed by sequencing the PCR amplicon obtained from genomic DNA using PCR primers SCO7671TF and SCO7669TR). Similarly the triple deletion mutant M1317 (Δ*sco7221*, Δ*sco7669*-*7671*, Δ*sco1206*-*1208*::*aac*(*3*)*IV*) was derived from M1316 using pIJ13002. Despite repeated attempts, it was not possible to remove the Apr^R^ cassette from M1317 using pIJ13008. Therefore, M1317 was confirmed by sequencing of the PCR product obtained from genomic DNA using PCR primers SCO1206TF and SCO1208TR. In each case the PCR fragment was cloned into pGEM^®^-T Easy and sequenced using M13 universal primers to verify that the expected deletions had been made.

**Table 3:** Primers used in this study

Primer	Sequence 5′–3′	Description
pIJ86F1	ACGCCTGGTCGATGTCGGAC	Sequencing primers for recombinant plasmids of pIJ86 and pIJ12477
pIJ86R2	TGCGGTCAGTGCGTGTGTCG	
SCO1206F1	ATCCCCAAGACCGAGGACTG	Sequencing primer for internal sequence of *sco1206*-*1207*-*1208*
SCO1206F2	AGGCCGTCATGGACCGCCAG	
SCO1206F3	TGTTCCACCTGCTGCTGAGC	
SCO1206F4	AGTGGTGAGCGGCCTGGTC	
SCO1206FBglII	AAAAA**AGATCT**CGCAAGCCTTCCGCGAGGCG	PCR of *sco1206*-*1207*-*1208* for cloning into pIJ12477
SCO1208RHindIII	AAAAA**AAGCTT**CTAACCGTGTCGCAGCGGCGTGAG	
SCO1206FSpe	CGCAAGCCTTCCGCGAGGCGAAAGCAGGCACGGTTCATG**ACTAGT**ATTCCGGGGATCCGTCGACC	Flanking primers for *sco1206*-*1207*-*1208* disruption cassette
SCO1208RNhe	GATCGCGCGGACGCTGGCCGGGATGCCGATCCGCTAACC**GCTAGC**TGTAGGCTGGAGCTGCTTC	
SCO1206TF	TCGAGCTGGCCAAGCTG	Test primers for verification of Apr^R^ cassette replacements and in-frame deleted regions
SCO1208TR	TGGAGTACGCGCAGACC	
SCO7221TF	AGTTGCATGGGTCACTGC	
SCO7221TR	ATGTACCGGCCCAGAGC	
SCO7671TF	GTTGCACCAGCCGATGG	
SCO7669TR	GTGAGTCGATGACTGTCGTGG	
SCO7221F1	AGTCGGTGCTCCGGCTGGAC	Sequencing primer for internal sequence of *sco7221*
SCO7221FBamHI	AAAAA**GGATCC**CCTCACCTGCTCCGCAGCAGACCC	PCR of *sco7221* for cloning into pIJ86 and pIJ12477
SCO7221RHindIII	AAAAA**AAGCTT**CTACAGCCACTCCCCTTCCAGAGCGGTGG	
SCO7221FSpe	CGCCGTGATACACGGCGAGCACTCCGTCGAGACCCGAGA**ACTAGT**ATTCCGGGGATCCGTCGACC	Flanking primers for *sco7221* disruption cassette
SCO7221RNhe	GTGGCGGTCAGGCCCGGGGCCGCCGCGTACAGGACGGCA**GCTAGC**TGTAGGCTGGAGCTGCTTC	
SCO7671FSpe	GTACGCACGGTAGTGGGGCCGGCGGCCGAGGAAGGCATG**ACTAGT**ATTCCGGGGATCCGTCGACC	Flanking primers for *sco7669*-*7670*-*7671* disruption cassette
SCO7669RNhe	GTCCGCTACGCGCGCGCCGCGCGGGGCCGGAACGGCTCA**GCTAGC**TGTAGGCTGGAGCTGCTTC	
SVEN5367F1	GGCCCGACACCGAGGACTG	Sequencing primer for internal sequence of *sven5367*
SVEN5367FBamHI	AAAAA**GGATCC**CGGGTGAGTGTGGGCGGCAGTTC	PCR of *sven5367* for cloning into pIJ12477
SVEN5367RHindIII	AAAAA**AAGCTT**TCAGGCGACGGACGTGCGGG	

Bolds indicate restriction sites

The three mutants were grown on SFM agar until sporulation. Spores were harvested and stored in 20 % glycerol at −20 °C.

### Construction of the type III PKS expression strains

*sco7221* (*gcs*) and *sco1206*-*sco1207*-*sco1208* (*rppA* operon) from *S. coelicolor* and *sven5367* (*rppA* homologue) from *S. venezuelae* were amplified from cosmids St2H12, 2StG58 and Sv3G11, respectively, using primers SCO7221FBamHI and SCO7221RHindIII, SCO1206FBglII and SCO1206RHindIII, and SVEN5367FBamHI and SVEN5367RHindIII, respectively (Table 3). The forward (F) primers were designed to include the ribosome binding sites of *sco7221*, *sco1206* and *sven5367*, respectively. The PCR products were cloned into pGEM^®^-T Easy and confirmed by restriction enzyme digestion and DNA sequencing using M13 universal primers and the internal primers SCO7221F1 for pGEM-T/*sco7221*, SCO1206F1, SCO1206F2, SCO1206F3 and SCO1206F4 for pGEM-T/*sco1206*-*sco1207*-*sco1208*, and SVEN5367F1 for pGEM-T/*sven5367* (Table 3). Two multi-copy expression vectors, each containing the constitutive ermE* promoter, were used; pIJ86 [48] was employed in M1152, while pIJ12477 [49] (a derivative of pIJ86 also conferring kanamycin resistance) was used in M1152 and in M1317. *sco7221* was sub-cloned as a *Bam*HI–*Hin*dIII fragment into both pIJ86 and pIJ12477, yielding pIJ86 + *sco7221* and pIJ12477 + *sco7221*, respectively. *sco1206*-*sco1207*-*sco1208* (*rppA* operon) were sub-cloned as a *Bgl*II–*Hind*III fragment into *Bam*HI plus *Hind*III cleaved pIJ12477, yielding pIJ12477 + *sco1206*-*sco1207*-*sco1208*. *sven5367* was sub-cloned as a *Bam*HI–*Hin*dIII fragment into pIJ12477 to obtain pIJ12477 + *sven5367*. Primers pIJ86F1 and pIJ86R2 (Table 3), which anneal 220 and 229 bp from the *Bam*HI and *Hind*III cloning sites, respectively, were used to verify the recombinant plasmids by PCR amplification and sequencing in addition to restriction enzyme digestion. For pIJ12477 + *sco1206*-*sco1207*-*sco1208*, primers pIJ86F1 and the internal primers SCO1206F1, SCO1206F2, SCO1206F3 and SCO1206F4 were also used for sequencing. The plasmids were then introduced into *E. coli* ET12567/pUZ8002 by transformation and transferred to the appropriate *Streptomyces* host by conjugation as described previously [43]. Exconjugants were selected on SFM agar containing 50 µg/ml apramycin or 50 µg/ml kanamycin as appropriate and 25 µg/ml nalidixic acid. Spore stocks were prepared on SFM agar containing the appropriate antibiotic for plasmid selection and stored in 20 % glycerol at −20 °C.

### Analysis of germicidin and flaviolin production

Cultures were grown for 5 days in 50 ml of GYM liquid medium, 5 ml of each culture supernatant lyophilized, the dried samples dissolved in 500 µl water and filtered through a VectaSpin Micro Polysulphone 0.2 µm column (Whatman). The samples were injected onto a Phenomenex Gemini-NX3u C18 110A, 150 × 4.6 mm column fitted to an Agilent 1100 HPLC system and analyzed using a linear methanol:water gradient in 0.1 % (w/v) formic acid as follows: 0 min, 10 % methanol; 1 min, 25 % methanol; 21 min, 100 % methanol; 25.50 min, 10 % methanol; 33 min, 10 % methanol, at a flow rate of 0.8 ml/min with UV absorbance monitoring at 280 nm. Fractions of interest were collected and analyzed by mass spectrometry (Thermo Scientific™). Germicidins were analyzed using positive ion mode mass spectrometry whereas flaviolin was analyzed using negative ion mode. Each experiment was carried out in duplicate. The mycelium from each 50 ml culture was harvested by centrifugation, dried and the dry weight determined. Relative levels of germicidin and flaviolin production were calculated by multiplying the average peak area (mAU) obtained from a 5 ml sample by ten and dividing by the average dry weight obtained from a 50 ml culture.

## Abbreviations

ACP: acyl carrier protein
Apr: apramycin
Gcs: germicidin synthase
Hyg: hygromycin
Kan: kanamycin
PKS: polyketide synthase
THN: 1,3,6,8-tetrahydroxynaphthalene
